# The Use of Consumer Wearable Physical Activity Monitors in Clinical Populations with Functional Limitations

**DOI:** 10.33696/rehabilitation.3.022

**Published:** 2021

**Authors:** Julian Martinez, Taylor M. Gordon, Scott J. Strath

**Affiliations:** Department of Kinesiology, University of Wisconsin-Milwaukee, Milwaukee, WI 53201-0413, USA

**Keywords:** Physical Activity, Activity Monitor, Fitbit, Arthritis, Multiple Sclerosis, Parkinson’s, Stroke

## Abstract

Functionally limiting health conditions have a high rate of prevalence worldwide and incur a significant amount of economic burden. Physical activity (PA) can prevent the onset of these conditions and alleviate economic burden by reducing symptoms, but a large portion of these individuals do not engage in health enhancing PA. Consumer wearable physical activity monitors (WPAM) are tools that have become increasingly popular within the past few years and could provide a means to improve PA levels for individuals with health conditions that cause functional limitations. This review reports on the validity of PA outcomes, feasibility and utility, and intervention/promotion effectiveness for consumer WPAM in functionally limited clinical populations. 2250 records from January 2018 to July 2021 were retrieved from PubMed, Web of Science, SPORTDiscus and CINAHL with 656 records being duplicates and 23 records passing a full-text article review. Studies included within the review looked at individuals with osteoarthritis, rheumatoid arthritis, axial spondyloarthritis, multiple sclerosis, Parkinson’s disease, ischemic stroke and peripheral arterial disease. The most popular brand of consumer WPAM was Fitbit. Validation studies for consumer WPAM were primarily focused on step counts showing overestimations for daily step counts and over- and under-estimations occurring within shorter time durations depending on step cadence. Wrist worn WPAM are the most feasible for functionally limited clinical populations with widespread utilization for associating clinically relevant outcomes with PA levels but they have limited validation to confirm their accuracy and precision in measurement. Interventions included used a mixture of a WPAM and other behavior change techniques to improve PA levels for clinical populations and show promising effectiveness. Future work is warranted on determining the validity of PA outcomes from WPAM determined to be feasible in select clinical populations and creating interventions looking at which features of a consumer WPAM intervention promote PA.

## Introduction

The prevalence of many musculoskeletal and cardiovascular diseases that cause limitations in physical functioning has increased globally [1] with age-adjusted rates of incidence rising for many of these diseases [2–5]. In the United States alone, chronic health conditions that limit physical function have an estimated economic impact of $200 billion per year [6]. There is evidence suggesting regular physical activity (PA) and exercise can alleviate symptoms and help prevent the onset of chronic conditions that cause functional limitations [7–11]. However, it has been observed in many epidemiological studies that individuals with chronic health conditions are significantly less active than their healthy counterparts [12,13]. For example, data from the Osteoarthritis Initiative in the United States show only 44.1% of men and 22.2% of women who are at risk or have knee osteoarthritis meet the 2018 Physical Activity Guidelines for Americans [14]. Given this, there is an enormous public health opportunity for investigating ways to increase PA in populations that have chronic health ailments that impact their level of physical functioning.

Wearable technology for health has been a growing market and social trend [15,16] that represents one avenue for measuring PA, increasing PA levels, and measuring clinically relevant outcomes. For measuring PA, consumer wearable physical activity monitors (WPAM) are low-cost devices that allow users to self-monitor changes in PA, which allow them to be easily adopted as facilitators of PA behavior change [17]. Previous research has been done in reviewing the literature of consumer WPAM for their validity and effectiveness as behavior change tools [18,19]. This mini-review aims to extend the previous research of consumer WPAM as a tool for measuring and promoting PA by reviewing the literature focused on clinical populations with functional limitations. Specifically, in this review we will examine the validity, feasibility and utility of use, and intervention/promotion effectiveness of WPAM for individuals with musculoskeletal, cardiovascular and neurological health conditions, and provide future recommendations to progress this line of scientific investigation.

## Methods

A small-scale systematic review was performed following the Preferred Reporting Items for Systematic Reviews and Meta-Analyses (PRISMA) 2020 guidelines [20]. Records were considered eligible for review if they met the following criteria: 1) used a consumer WPAM as a tool to measure a PA outcome, 2) recruited participants aged 18 years or older that were not receiving in or outpatient care, and 3) recruited participants that were either diagnosed or self-reported as having a musculoskeletal, cardiovascular or neurological condition that is associated with less physical functioning, including osteoarthritis (OA), rheumatoid arthritis (RA), multiple sclerosis (MS), Parkinson’s disease (PD), ischemic stroke and peripheral arterial disease (PAD). Records obtained were grouped by health condition. As there is no clear definition of what constitutes a consumer WPAM as opposed to a research-grade WPAM, such as an ActiGraph (AG) GT_3_X+ or activPAL (AP) 4, online marketplaces and product descriptions were searched to determine if a WPAM was marketed towards a consumer. Four databases were searched (PubMed Central, Web of Science, SPORTDiscus and CINAHL) with the following search terms: (arthritis OR “rheumatoid arthritis” OR osteoarthritis OR “multiple sclerosis” OR “ms” OR parkinsons OR parkinson’s OR “parkinsons disease” OR “parkinson’s disease” OR stroke OR “cerebral palsy” OR fibromyalgia OR “post-polio syndrome” OR “spinal cord injury” OR “traumatic brain injury” OR neurodegenerative ) AND ( “physical activity” OR “exercise” OR “sedentary behavior” OR sedentary OR “step count” OR “energy expenditure” OR intensity ) AND ( “activity monitor” OR “activity tracker” OR fitbit OR jawbone OR garmin OR nokia OR misfit OR sensewear OR pedometer OR accelerometer OR “wearable device” OR “wearable” OR “smart device” OR “consumer” OR “apple watch” ) NOT ( child OR children OR youth OR infant OR athlete ). Filters used were a publication date between January 1^st^, 2018 and July 20^th^, 2021 and the article being written in English.

After search results were received, a simple R script was written to 1) combine PubMed results with abstracts and 2) remove duplicates of records by comparing lowercased titles between records with special symbols removed. Titles and abstracts of records were then screened by authors JM and TG independently. A record was excluded if it did not meet the eligibility criteria, only included an abstract, was a proceedings paper from an academic meeting, was a case study, or systematic review. If a record was reporting on a re-analysis or a secondary analysis of a study that took place before 2018, it was only included if the study took place after January 1^st^, 2015 in order to focus on new research in the field. Studies included in prior systematic reviews were searched as another method for identifying potential records. If a record’s title or abstract mentioned the use of an accelerometer, a sensor typically used to estimate PA, within the abstract without a brand or if it was unclear a consumer WPAM was used to assess a PA outcome, the full-text article for the record was sought for retrieval and then evaluated to determine if eligibility criteria were met. If there was still uncertainty by a reviewer on the eligibility of a record, authors JM, TG and SJS reached a consensus on the eligibility of the record. Records that met the eligibility criteria then had the full-text articles read entirely by authors JM and TG for further screening. Once records were fully screened, the following data was retrieved from the articles: purpose and/or hypothesis of the study, participant size and characteristics, study design, consumer WPAM used, and PA outcome measure from WPAM. Included records were then grouped into one of three categories based on the study purpose/hypothesis: validation of WPAM PA estimates, feasibility & utility of WPAM within observational studies and WPAM intervention use for PA behavior change as either the motivational tool for change or as the measurement tool for change.

## Results

**Figure 1.**
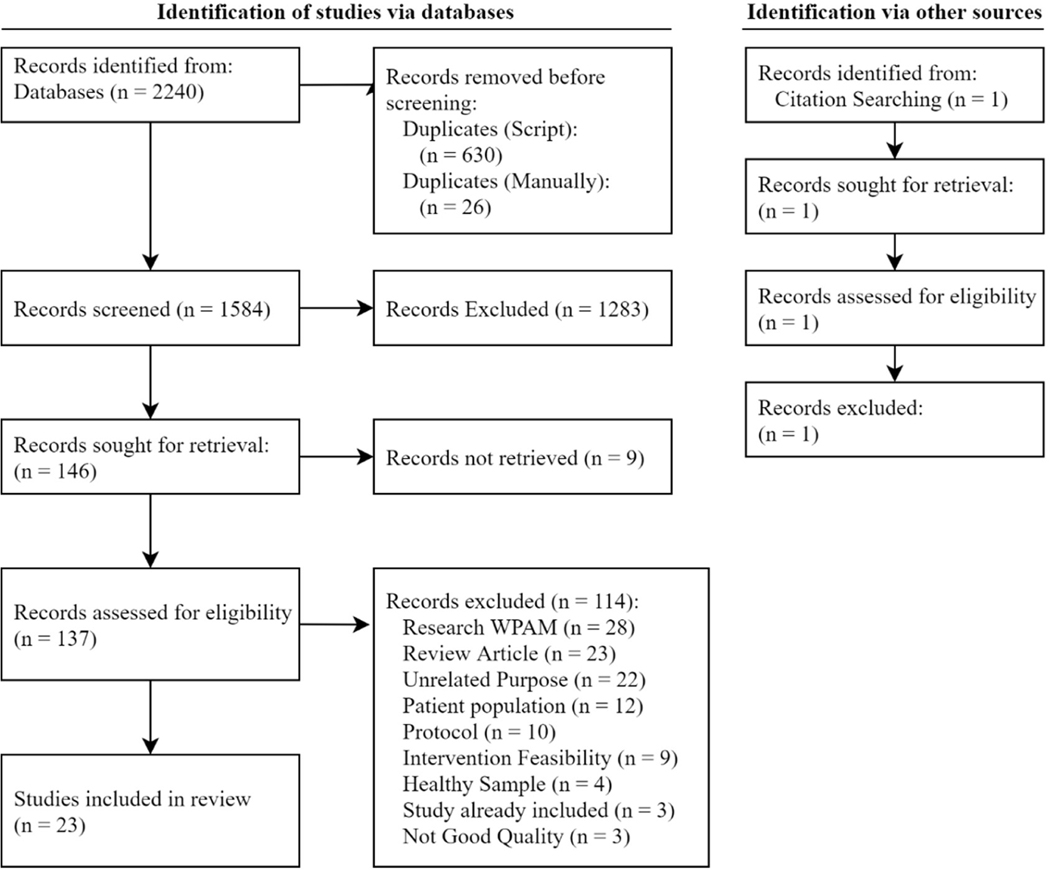
Modified Preferred Reporting Items for Systematic Reviews and Meta-Analyses (PRISMA) Flow diagram of screening process. WPAM: Wearable Physical Activity Monitor.

A flowchart of identification, screening and inclusion is shown in Figure 1. 2250 records were retrieved from database searching with 656 records being duplicates. 146 records passed the screening process with 23 records passing a full-text article review. Out of the 23 studies, 7 were focused on validating PA measures from a consumer WPAM, 13 explored the feasibility of implementing a WPAM for a specific population and utilizing a WPAM within observational studies to determine associations related to PA and 3 focused on using a WPAM for PA tracking and behavior change within an intervention. Table 1 provides characteristics of included studies and Table 2 lists the PA outcomes, price and features for consumer WPAMs included in the review.

## Discussion

The purpose of this mini-review is to synthesize the research within the past few years on the validity, feasibility and utility of use and intervention/promotion effectiveness of consumer WPAM in functionally limited clinical populations. Understanding the synthesis of results within this review requires knowledge on the complex nature of PA behavior, sensors included in WPAM that are used to estimate PA, estimates of PA outcomes that are outputted from these sensors and gold-standard methods for measuring these outcomes. These topics have been reviewed in detail previously [18,21–23]. Briefly, PA constitutes “any bodily movement produced by the contraction of skeletal muscles that results in energy expenditure (EE)” [24]. This bodily movement is complex and can be broken down into many different components such as frequency of movement, intensity of movement, mode of movement, and duration of movement. Sensors, such as accelerometers, altimeters, gyroscopes and magnetometers, are used to measure these components of PA for estimating EE-related outcomes. These EE-related outcomes include calories spent during a duration of PA, steps per day, time spent in physiological postures (sitting vs. standing) and time spent in different PA intensity categories. Sedentary, light, moderate, vigorous and moderate-to-vigorous physical activity (MVPA) are standard intensity categories with time spent in each category having implications for health. Research-grade WPAM are typically used for their accuracy in estimating these EE-related outcomes, ability to download data directly from the monitoring device and transparency in data algorithms whereas consumer WPAM are convenient, low-cost measuring tools focused primarily on assessing and promoting PA with industry or manufacturer propriety algorithms.

### Validity of consumer wearable physical activity monitors in functionally limited clinical populations

#### Minutes within an intensity category:

Two studies included within this review examined consumer WPAM estimated minutes within a PA intensity category while most studies were focused on steps. Semanik et al. [25] focused on office employees living with chronic knee symptoms to determine the validity of average daily time spent in different PA intensity categories from the Fitbit Flex compared with research grade AG GT_3_X+ estimations. Specifically, time spent in light intensity activity and bouts of 10-minutes or more in moderate, vigorous and MVPA were compared. Bland-Altman plots indicated that the Fitbit Flex underestimated light intensity minutes when AG estimates were low and overestimated light intensity minutes when AG estimates were high. For MVPA, the Fitbit Flex systematically overestimated minutes with the magnitude of overestimation increasing as AG estimations increased. Collins et al. [26] looked at a similar population for determining the validity of PA intensity minutes from the Fitbit Charge 2 compared to a research-grade AG GT_3_X+ estimates for individuals with knee OA. Results showed that the Fitbit Charge 2 overestimated average daily sedentary time by 37% (2.1 hours).

#### Average steps per day:

Collins et al. [26] also compared average daily step counts between the two monitors and found the Fitbit Charge 2 to overestimate steps per day by as much as 39% (1,648 steps). An inter-class correlation coefficient (ICC) of 0.602 was also seen where it is generally considered that ICC “values less than 0.5, between 0.5 and 0.75, between 0.75 and 0.9, and greater than 0.90 are indicative of poor, moderate, good, and excellent reliability, respectively”. [27] Another study that compared average step counts per day, this time looking at Fitbit Flex and Fitbit Flex 2 estimates for people with MS to a research-grade AG GT_3_X+, found that both of the consumer WPAM overestimated steps per day. The Fitbit Flex overestimated an average of 873 steps per day while the Fitbit Flex 2 overestimated 808 steps per day with the magnitude of overestimation for both monitors increasing for individuals with higher Expanded Disability Status Scale scores [28]. This study also compared total step counts from the Fitbit Flex to directly observed hand-tallied step counts during a 2-minute walk test and found no systematic bias. This result from a short duration walk contradicts the results on the validity of WPAM estimates for average steps accumulated during the course of a day. This discrepancy is likely due to the total day values being compared with a research grade WPAM and not being compared to a criterion standard hand-tallied count.

#### Total steps within short durations:

Further exploring the validity of consumer WPAM step counts in shorter time durations, Lai et al. [29] compared consumer WPAM total step counts during timed walks to manually tallied criterion step counts for people with PD. The authors found the Fitbit One to be the most accurate and precise when participants were asked to walk for 6 minutes over normal ground and on a treadmill at their self-selected pace. Looking at shorter walking durations, Lamont et al. [30] had participants with PD, while wearing a Fitbit Charge HR and Garmin Vivosmart, perform six 2-minute walks at their self-selected pace and at cadences of 60, 80, 100, 120 and 140 beats per minute. Using step count estimates from a research-grade AP as the criterion, both the Fitbit Charge HR and Garmin Vivosmart had an average percent error ≤ 3% but had greater average percent errors during the walks at pre-determined cadences. Further exploring the accuracy of step counts for people with PD, Wendel et al. [31] looked at the accuracy of consumer WPAMs to manually tallied criterion steps while participants walked in simulated house and obstacle courses. No WPAM had an ICC above 0.17 for the household course while the Fitbit Zip had an ICC of 0.58. In our review, only one included study looked at the accuracy of a consumer WPAM to manually tallied criterion step counts for post-stroke individuals in a variety of walking settings [32]. The authors found that a Fitbit Zip placed on the non-paretic ankle for individuals were accurate in a laboratory setting but errors increased within a mall circuit that included stairs and ramps.

### Summary:

A majority of validation studies in the past couple of years have been focused on step counts estimates from consumer WPAMs, showing them to differ in accuracy depending on whether total step counts within a laboratory setting or average daily step counts are the outcome of interest. No studies looking at the validity of average daily step counts have used a true criterion or gold-standard measure, or a comparison measure that has been shown to be accurate and precise compared with a gold-standard criterion measure. For example, the research-grade WPAM StepWatch, which has shown to be the most accurate in estimating step counts compared to direct observation [33], could be used in lieu of research-grade monitors for naturalistic or field-based daily validation studies. Alternatively, comparing the StepWatch to a research-grade WPAM in clinical populations with functional limitations would extend the utility of these monitors to be used as comparison standards to consumer WPAMs.

### Feasibility and utility of implementing consumer wearables in clinical populations

#### Feasibility:

In the past few years, a number of qualitative and mixed-method studies have looked at the feasibility of implementing WPAMs for clinical populations. Beukenhorst et al. [34] conducted semi-structured interviews with 26 older individuals who wore a Huawei Watch 2 smartwatch for 3 months every day. An app developed for the study was also installed on the watch and asked participants questions relating to pain and knee OA symptoms. Themes that arose from the interviews were the ease of using the smartwatch, motivation to see a relationship between their pain levels and activity levels (determined by step count), and doubts about the smartwatch’s battery life and step count accuracy. Beukenhorst and colleagues also found the median daily wear time over the 90 days to be approximately 11 hours and that the watch was worn for 73% of the study period [34]. Manini et al. [35] also developed an app for the Samsung Gear S3 smartwatch that asked older individuals with knee OA about pain throughout the day. Instead of having individuals wear the smartwatch for a period of time, focus groups were set up with participants where the smartwatch + app was shown to the group first and questions were asked afterwards about their thoughts on the watch. Themes that arose from the focus groups were a desire for customizing how the watch would alert them and the ability to provide more detailed information than the questions provided on the smartwatch app. For individuals with RA and/or axial spondyloarthritis, the ActConnect study gave participants a Withings Activité Pop watch to wear every day for 3 months [36]. From 177 participants, the activity monitor was worn for a mean time of 79 days (88%) over the 90-day period. From a questionnaire asked at the end of the period, 97% of participants considered daily use of the watch acceptable and 63% of participants even considered keeping some sort of activity tracker on after the end of the study. Focusing on people with MS, Fortune et al. [37] purposefully recruited 15 participants after the end of a randomized control trial to be part of semi-structured interviews. Purposeful recruitment was done to get a wide range of experiences (e.g. high and low activity engagement, different life commitments, gender) during the interview. Themes that arose from the interview regarding their experience of the hip-worn Yamax SW200 Digiwalker were increased activity awareness through the pedometers digital display, objective numeric feedback from a monitor that leads to increased motivation, and placement difficulties that led through decreased confidence in the accuracy of pedometer readings.

Looking at quantitative measures of feasibility for consumer WPAMs, Block et al. [38] examined wear-time compliance during the FITRiMS study in which 95 individuals with MS were recruited to wear the Fitbit Flex every day for one year. Of the 79 individuals that were retained to the end of the study, an average of 3 valid weeks of average daily step counts were available each month in which a valid week consisted of 3 or more valid days and a valid day consisted of 128 steps or more. In other clinical populations, Pradhan et al. [39] saw that 30 individuals with PD found the Fitbit Flex easy to use for 14 full days with 66% of individuals stating increased motivation. Katzan et al. [40] found that 15 participants, whom were required to wear the Fitbit Charge HR after hospital discharge for ischemic stroke, wore the WPAM for 83.6% of a 90-day period and Elmagboul et al. [41] saw that a Fitbit Charge HR 2 was worn for 60.5% of the time, determined from when there was available heart rate, step count or sleep data in a day, out of an average of 6572 days for individuals who experienced gout flares.

#### Utility:

Consumer WPAMs have also been utilized to measure prospective and cross-sectional associations of PA with functional disability and other chronic conditions for clinical populations. The ActConnect study explored factors associated with lower PA in individuals with RA and axial spondyloarthritis using a smartwatch [36]. For people with MS, a Beurer AS 80 and Yamax SW 200 Digiwalker were used to determine cross sectional associations of serum vitamin D levels and activity capacity with daily PA levels [42,43]. For individuals who experienced gout flares, were in the early stages of PD, were stroke survivors, and experience fibromyalgia, step counts and time spent in MVPA from a Fitbit device worn for ≥ 7 days was used to measure PA levels [39,41,44–46].

#### Summary:

Qualitative and quantitative measures of feasibility and utilization in observational studies for determining PA associations have shown there is a great interest in using wrist-worn WPAMs to measure PA levels. Only one hip-worn consumer WPAM included has been examined for feasibility and utilized for a functionally limited clinical population. This follows the trend of wrist-worn monitors increasing in popularity these past few years, with many new monitors being able to function as a smartwatch to tell time, answer calls and connect with smartphones for increased functionality. Despite the shift in using more wrist worn consumer WPAM, stemming from ease of wear, high compliance and their dual functionality, there is limited research on their validity for measuring PA outcomes. As discussed previously, the majority of validation work in this area has been focused on older consumer WPAM and not on newer smart watch WPAM technology

### Intervention/promotion use of consumer wearables in clinical populations

#### Interventions included:

Despite the increasing use of consumer WPAMs for measuring associations of PA with clinically relevant outcomes for individuals with a functionally limiting condition, there is a paucity of research in the past few years in determining the effectiveness of interventions for community-dwelling individuals who have health conditions impacting their physical functioning. Within the limited work completed in this area, Li et al. [47] conducted a 12-week multifaceted randomized control trial in individuals with knee OA. 51 individuals were randomized to an immediate group and delayed group in which the delayed group received the intervention 14 weeks after group allocation. The intervention included 4 biweekly phone calls from physical therapists to monitor progression in achieving SMART goals, a Fitbit Flex 2 to track PA and a web-based app developed for the study that interacted with the Flex 2 to monitor progress towards goals. After 8 weeks of the intervention, physical therapists stopped counseling with participants for weeks 9–12. Difference in time spent performing MVPA, measured by a SenseWear Mini WPAM for 7 days at week 13, between the immediate and delayed group was the primary outcome. Results showed that the immediate group had significantly increased their PA by 13.1 more minutes of MVPA per day compared with the delayed group.

In another study focusing in PD patients, Ellis et al. [48] recruited 51 individuals to participate in a 12-month randomized control trial that involved a mHealth group and an active control group to see if increases in daily step counts improved walking capacity and health-related quality of life. Both groups were given a consumer WPAM to track activity levels (Fitbit Zip and Omron HJ-113 for mHealth and active control group, respectively) with the mHealth group having more accessible communication with a physical therapist and action plans through dedicated app developed for the iPad. Results showed that there was no significant difference in step counts or time spent in MVPA between and within groups. However, individuals within the mHealth group who achieved less than 7500 steps/day at baseline did see a significant increase of 763 steps/day and 55.7 minutes of moderate intensity activity which was not seen in the active control group. As for walking capacity and health-related quality of life, only the mHealth group saw significant improvements.

Finally, McDermott et al. [49] recruited 200 individuals with peripheral arterial disease (PAD) for a 9-month randomized control trial to improve 6-minute walking test (6MWT) distance, Patient-Reported Outcomes Measurement Information System (PROMIS) scores, and total average steps per day. The intervention was homebased and included behavioral change techniques that followed participant feedback from focus groups, social cognitive theory concepts and a Fitbit Zip consumer WPAM. Results showed no significant improvement in 6MWT distance or step counts within the intervention group or compared to the usual care group, but there was a significant improvement in PROMIS pain interference scores for the usual care group. The authors noted that total day estimates from the consumer WPAM may have influenced participants to increase overall activity whereas the intervention was focused on increasing bouts of walking throughout the day. They point to previous literature showing that increasing overall activity level does not increase walking endurance for individuals with PAD.

#### Summary:

The few studies included have used a consumer WPAM in conjunction with other behavior change techniques, such as direct communication with physical therapists. Results from these reviewed interventional studies show promising results for individuals with knee OA and PD but not for individuals with PAD. As the number of interventions focused on community-dwelling clinical populations within the past few years is small, there is a need to increase research in this area to fully explore the ability of consumer WPAM to be used as a tool to promote and increase PA behavior and health outcomes.

### Recommendations for Future Research

The authors suggest possible avenues of research that can be explored for the use of consumer WPAMs for clinical populations.

- Coinciding with Strath et al. [18], the consumer WPAM industry should reach out to researchers specialized in objective measurements of PA to improve estimates of PA from consumer WPAMs.
- Validation studies would benefit from following a framework similar to the one suggested by Keadle et al. [23]. Specifically, the use of gold-standard measures as the criterion should be used while validating a consumer WPAM from a laboratory setting to more free-living settings in clinical populations.
- To our knowledge there has been no study to determine the accuracy and precision of step count estimations from research-grade WPAMs in free-living settings for individuals with functional limitations. As daily step counts from consumer WPAMs have been a PA outcome in many of the studies within this review and included validation studies used a research-grade WPAM in lieu of an established criterion, further work is needed in validating research-grade WPAMs before they can be used as a criterion for validating daily step counts from consumer WPAMs.
- The feasibility of smartwatches and apps developed for them have been researched for select clinical populations. Determining the validity of PA outcomes from smartwatches and utilizing them more in observational and interventional studies is an area of research that has yet to have been fully explored.
- Features specific to consumer WPAMs that promote behavior change should be examined in functionally limited clinical populations. This will allow researchers and the consumer WPAM industry to determine what features are most effective for behavior change.

## Conclusion

The use of consumer wearables for clinical populations is an area of research that will continue to grow. Fitbit monitors have been researched in all manners for a variety of clinical populations while smartwatches are beginning to see more utilization in observational and validation studies. With the smartwatch market expected to grow from $20.64 billion in 2019 to a projected $96.31 billion by 2027 [50], there is great potential for smartwatches to be included in future studies involving clinical populations. Regardless, the continued use of consumer WPAM for measuring PA outcomes and improving health-related factors for clinical populations continues to be a growing field with many exciting possibilities for use.

## Figures and Tables

**Table 1:** Study characteristics.

Study Category	Author	Population	Device(s)	Main Outcomes	Results
Validation	Semanik et al. [25]	Arthritis (n = 35) Illinois, USA	Fitbit Flex	Daily time in Light and MVPA compared to AG GT_3_X+ hip.	−37.7 minute bias for light intensity; 18.7 minute bias for MVPA. Under and overestimations increase as criterion estimate increases.
	Collins et al. [26]	Osteoarthritis (n = 15) Massachusetts, USA	Fitbit Charge 2	ICC of step counts against AG GT_3_X+ hip and % bias of daily step counts	ICC = 0.602. 39% bias for step counts (5,084 steps from criterion);
				Sedentary time to AG GT_3_X+ hip.	37% bias for sedentary time (2.1 hours criterion)
	Block et al. [28]	Multiple Sclerosis (n = 61) California, USA	Fitbit Flex	Absolute step count to manual counts during 2MWT;	No significant bias reported although the Fitbit Flex overestimated.
		(n = 36)	Fitbit Flex Fitbit Flex 2	Flex 2 and Flex daily step counts to AG GT_3_X	Fitbit Flex2: Significant bias of 808 steps/day. Fitbit Flex: Significant bias of 873 steps/day.
	Lai et al. [29]	Parkinson’s (n = 31) Alabama, USA	Fitbit One Fitbit Charge 2 Garmin Vivosmart 3	Absolute step counts over a indoor course for 6 minutes to manual counts	ICC ≥ 0.97 for both Fitbit One and Garmin Vivosmart 3. ICC of 0.47 for Fitbit Charge HR 2.
				Absolute step counts during treadmill walking for 6 minutes to manual counts	Fitbit One: ICC of 0.98 Fitbit Vivosmart 3: ICC of 0.67 Fitbit Charge HR 2: ICC of 0.27
	Lamont et al. [30]	Parkinson’s (n = 33) Queensland, Australia	Fitbit Charge HR Garmin Vivosmart	Absolute step counts over 2-minute walking tests at a self-selected pace, 60, 80, 100, 120 and 140 steps/minute to AP	Both Monitors: APE < 3.0% and ICCs ≥ 0.88 at self-selected pace. Both Monitors: APE ≥ 37.2% & ICC = 0.36 at 60 steps/minute. Fitbit Charge HR: APE of 3.5–17.6% & ICC of 0.18–0.37 for other cadences. Garmin Vivosmart: APE < 5.0% & ICC of 0.68–0.89 for other cadences
				Absolute step counts during a 500m outdoor walking course (slopes, grass, stairs, crowds of people) at a self-selected pace to AP	Fitbit Charge HR: APE = 1.5% & ICC = 0.94 Garmin Vivosmart: APE = 1.9% & ICC = 0.97
	Wendel et al. [31]	Parkinson’s (n = 35) Massachusetts, USA	Fitbit Surge Fitbit Zip Jawbone Up Move Jawbone Up 2	Absolute step counts during a 2-minute walk test at a comfortable and fast pace to manual counts	Fitbit Zip: ICCs ≥ 0.90 & Bias ≤ 2.15 steps Comfortable Pace: Lowest ICCs & Bias seen for Surge and Up 2 (0.38, 0.10 & −14.28, −4.00 steps, respectively). Fast Pace: Lowest ICCs & Bias seen for Surge and Up 2 (0.13, −0.02 & −23.03, −7.56 steps, respectively).
				Absolute step counts during a household simulated course and obstacle negotiation course to manual counts	Household: ICCs ≤ 0.17 & Bias ≥ −22.76 steps for all WPAMs Obstacle: Fitbit monitors had an ICC of 0.41–0.58 & Bias of −4.27 to −6.39 steps. Jawbone monitors had an ICC of 0.05 & Bias of −4.88 to −12.61 steps
	Duclos et al. [32]	Stroke (n = 17) Quebec, Canada	Fitbit One (non-paretic leg ankle)	Absolute step counts during 6-minute walk test, and circuit inside mall to manual counts.	APE = 0.50%
		(n = 13)	Fitbit One (hip)	Absolute step counts during 6-minute walk test, and circuit inside mall to manual counts.	APE = 2.67%
Feasibility	Beukenhorst et al. [34]	Osteoarthritis (n = 18) Manchester, UK	Huawei Watch 2	1 interview at baseline, 1 interview after wearing WPAM for 90 days. Interviews were semi-structured and developed from techno-utopian and critical approaches towards self-tracking.	Baseline Interviews: Motivation for participation revolved around yearning to learn more about relationship between PA and pain. Concern about operating smartwatch was present. End Interviews: Smartwatch was easy to use. Battery life and step counts restarting after a recharge were causes for frustration. There was enthusiasm for recording pain levels using smartwatch.
	Manini et al. [35]	Osteoarthritis (n = 19) Florida, USA	Samsung Gear S3	Focus groups following a semi-structured interview format consisting of questions related to impressions of smartwatch technology, ecological momentary assessment approach from smartwatch app on patient-reported outcomes (PRO)s and potential future improvements.	A desire for customizing when to be notified for PRO outcomes and how to respond (i.e. more detailed information than app allowed) appeared as a theme. Recommendation for assessments to occur throughout the day and then a summary assessment at the end of the day.
	Jacquemin et al. [36]	Rheumatoid Arthritis; Axial Spondyloarthritis (n = 177) Paris, France	Withings Activité Pop Watch	Adherence to wearing WPAM over 90 days.	WPAM worn 88% of days, 78.5% still wore smartwatch at end of study.
		(n = 171)		Acceptability questionnaire, inquiring about discomfort, security and utility of smartwatch, administered at the end of wearing WPAM for 3 months.	Out of a score of 10 (10 = Totally acceptable), a mean score of 8.5 was answered for acceptability of smartwatch. 63% said they will continue to wear the watch most of the time after the study end and 63% thought the watch allowed them to increase their PA. 17% needed help with the watch and 30% thought the watch underestimated PA.
	Fortune et al. [37]	Multiple Sclerosis (n = 15) Southeast England	Yamax SW-200 Digiwalker	One-to-one semi-structured interviews on engagement with PA and perspectives of WPAM.	Themes: A raised consciousness of PA levels, step goals are not abstract anymore, step counts were motivational for increasing PA. However, the accuracy of the WPAM was questioned and when it was inaccurate, demoralizing for participants. WPAM placement on hip caused difficulties for usability.
	Block et al. [38]	Multiple Sclerosis (n = 79) California, USA	Fitbit Flex	Valid days (at least 128 steps) of wearing WPAM for 12 months.	3 valid weeks (3 valid days or more) of average daily step counts per month. Participants with longer Timed-Up-And-Go times, greater disability and more pain only had one valid week of data per month.
	Pradhan et al. [39]	Parkinson’s (n = 30) Washington, USA	Fitbit Charge HR	Questions regarding the effort to learn how to use WPAM, satisfaction from using WPAM, and if WPAM provided motivation to be more active at the end of a 14-day wear period.	On a scale from 1 to 5 (5 = easy to use), an average score of 4.3 was reported for effort on learning WPAM use. On a scale of 1 to 5 (5 = very satisfied), an average score of 4.1 was reported on WPAM satisfaction. 66% of individuals reported increased motivation to be active.
	Katzan et al. [40]	Stroke (n = 15) Ohio, USA	Fitbit Charge HR	Participant adherence (days with ≥ 100 steps) during a 90-day wear period	Participants wore WPAM 83.6% of time.
	Elmagboul et al. [41]	Gout Flares (n = 33) Alabama, USA	Fitbit Charge 2	WPAM compliance in 4 categories: “Compliant wear with sleep” = Worn 80% of 1440 minutes, “Compliant wear without sleep” = 80% of 960 minutes, “No health tracker data” = no sensor data and “Partial wear” = all other patterns. A minute of wear time is defined as any minute where heart rate, step count or sleep data was available.	Out of 6572 days of collected data, 3978 days met a compliant wear pattern. Of 3978 days, 68% were classified as Compliant wear with sleep, 7% as Complaint wear without sleep and 25% as Partial wear.
Utility	Jacquemin et al. [36]	Rheumatoid Arthritis; Axial Spondyloarthritis (n = 157) Paris, France	Withings Activite Pop Watch	Comparison of step counts over 90-day wear period between rheumatoid arthritis and axial spondyloarthritis participants.	No significant difference was found between the two clinical populations over the 3 months.
				Description of PA levels for participants over 90 days.	Partitioning the participants into 3 clusters of homogeneous activity (low, moderate and high), 54.1% of participants had a low activity level, 42.7% had a moderate activity level and 3.2% had a high activity level.
	Bauer et al. [42]	Multiple Sclerosis (n = 38) Innsbruck, Austria	Beurer AS 80	Association between WPAM-defined activity time and 25-hydroxyvitamin-D3 levels over a 14-day wear period.	A weak correlation (0.221) was found between WPAM-defined activity time and vitamin D levels.
	Ryan et al.[43]	Multiple Sclerosis (n = 52) England	Yamax SW-200 Digiwalker	Associations between 6-minute walk test distance, walking ability assessed from Twelve Item Multiple Sclerosis Walking Scale and average daily steps over 6 days.	An increase of 10 meters during the 6-minute walk test was associated with a significant increase of 130 steps/day and a 1 point increase on the Twelve Item Multiple Sclerosis Walking Scale was associated with a significant increase of 87 steps/day.
	Pradhan et al. [39]	Parkinson’s (n = 30) Washington, USA	Fitbit Charge HR	Associations between average daily step counts, 10-meter walk times, balance and disease severity over 14 days.	There was a moderate correlation of step counts with self-selected and fast paces for the 10-meter walk (−0.60 and −0.64, respectively). A low significant correlation was found with balance scores (0.38) and no correlation was found with disease severity.
	Elmagboul et al. [41]	Gout Flares (n = 33) Alabama, USA	Fitbit Charge 2	Associations of average daily step counts with days of gout flares and days without gout flares.	A non-significant decrease of 396 steps/day were seen on days of gout flares compared to days without gout flares.
	Sasaki et al. [44]	Stroke (n = 22) Hyōgo Prefecture, Japan	Fitbit One	Association between average daily steps with EuroQoL 5-dimension 3 level health utility scores.	A significant positive correlation of 0.466 was found between average step counts and health utility score.
	Kanai et al.[45]	Stroke (n = 50) Hyōgo Prefecture, Japan	Fitbit One	Associations between average daily steps and MVPA to EuroQoL 5-dimension 3 level health utility scores over 7 days.	Multiple linear regression analyses showed the health utility score was significantly associated with an increase of steps but not an increase in MVPA.
	Lazaridou et al. [46]	Fibromyalgia (n = 107) Massachusetts, USA	Fitbit Flex	Associations between daily step counts, pain intensity and pain catastrophizing associations among daily pain symptoms, catastrophizing, and physical activity in patients with FM	Significant bivariate correlations were found between average state pain catastrophizing, pain intensity and step counts
Intervention	Li et al. [47]	Osteoarthritis (n = 51) British Columbia, Canada	Fitbit Flex 2	Mean daily MVPA over a 7-day period assessed by SenseWear Mini	A significant mean increase of 13.1 minutes in MVPA was observed in the primary group compared to a delayed intervention group.
	Ellis et al. [48]	Parkinson’s (n = 44) Massachusetts, USA	Fitbit Zip Omron HJ-113	Mean daily step counts and time spent in moderate intensity stepping over a 7-day period assessed by StepWatch.	A non-significant increase of 102.6 steps was observed for the intervention group. The increase was not significantly different from the active control group. A non-significant increase of 17.4 minutes in moderate intensity PA was observed for the intervention group. The increase was not significantly different from the active control group.
	McDermott et al. [49]	Peripheral Arterial Disease Illinois, Minnesota & New York, USA	Fitbit Zip	Change in 6-minute walk distance and average total step counts/day as assessed by an AG monitor.	A non-significant increase of 5.5 meters was observed for the intervention group. The increase was not significantly different from the usual care group. A non-significant decrease of 494 steps/day was observed for the intervention group. The decrease was not significantly different from the usual care group.

AG: Actigraph; ICC: Intra-class Correlation Coefficient; AP: activPAL; APE: Absolute Percentage Error; PA: Physical Activity

**Table 2.** Consumer Wearable Physical Activity Monitor characteristics taken from manufacturer website, user manuals and studies included within the review. Monitors that were not available for purchase from the brand manufacturer’s website were deemed as unavailable.

Brand (Website)	Monitor Name (Type)	Sensor(s)	Battery Life	Attachment	Dimensions (height × width × depth) (cm)	Market Price ($)	PA Outcomes	Water Resistance
Beurer (https://www.beurer.com)	AS 80 (AT)	Not Reported	Not Reported	Wrist with band	25.4×1.8×1.1	Unavailable	Steps Calories Distance Active Minutes Sleep	Splash Proof
Fitbit (https://www.fitbit.com)	Charge HR (AT)	Triaxial accelerometer Altimeter Optical Sensor (photoplethysmography)	5 days	Wrist with band	Not Reported	Unavailable	Steps Calories Distance Heart Rate Floors Active Minutes Sleep	Splash Proof
	Charge 2 (AT)	Triaxial accelerometer Altimeter Optical Sensor (photoplethysmography)	5 days	Wrist with band	Not Reported	Unavailable	Steps Calories Distance Heart Rate Floors Active Minutes Sleep	Splash Proof
	Flex (AT)	Triaxial accelerometer	Up to 5 days	Wrist with band	14–20.9×1.4 cm	Unavailable	Steps Calories Distance Active Minutes Sleep	Water-resistant
	Flex 2 (AT)	Triaxial accelerometer	Up to 5 days	Wrist with band	1.1 width	Unavailable	Steps Calories Distance Active Minutes Sleep	Water resistant up to 50 meters
	One (AT)	Triaxial accelerometer Altimeter	Up to 10 days	Clip	4.8 × 1.9 × 1.0 cm	Unavailable	Steps Calories Distance Sleep	
	Surge (AT)	Triaxial Accelerometer Altimeter Triaxial gyroscope Triaxial magnetometer GPS Optical Sensor (photoplethysmography)	7 days; 10 hours with GPS	Wrist with band	1.0 × 3.5 cm	Unavailable	Steps Calories Distance Heart Rate Floors Active Minutes Sleep	Splash Proof
	Zip (AT)	Triaxial Accelerometer	6 months	Clip (pocket, waist)	3.8 × 2.8 × 1.0	Unavailable	Steps Calories Distance	Splash Proof
Garmin (https://www.garmin.com)	Vivosmart (AT)	Not Reported	Up to 3 days	Wrist	Not Reported	Unavailable	Steps Calories Distance Heart Rate Active Minutes Sleep	Water resistant Up to 10 meters
	Vivosmart 3 (AT)	Not Reported	Up to 5 days	Wrist	1.9 × 1.0 × 2.0 cm	$119.99	Steps Calories Distance Heart Rate Floors Active Minutes Sleep	Water resistant up to 50 meters
Huawei (https://consumer.huawei.com/)	Watch 2 (SW)	Not Reported	Not Reported	Wrist with band	Not Reported	Unavailable	Steps Calories Distance Heart Rate	Water resistant up to 1.5 meters
Jawbone (https://www.jawbone.com)	Up 2 (AT)	Triaxial Accelerometer	Not Reported	Wrist with band	Not reported	Unavailable	Steps	
	Up Move (AT)	Triaxial Accelerometer	9.21 × 3.18 in	Clip	Not reported	Unavailable	Steps	
Withings (https://www.witliings.com)	Activite Pop Watch (AT)	Triaxial Accelerometer	8 months	Wrist with band	3.7 diameter	Unavailable	Steps Calories Distance Sleep	Water resistant up to 50 meters
Omron (https://omronhealthcare.com)	HJ-113 (Pedometer)	Pedometer	6 months	Clip (pocket, waist)	6.7× 4.8 × 1.6	Unavailable	Steps Calories Distance	None
Samsung (https://www.samsung.com)	Gear S3 (AT)	GPS Accelerometer Barometer Gyro Sensor Heart Rate Sensor Ambient Light Sensor	Up to 3 days	Wrist	4.6 × 4.9 × 1.3	Unavailable	Steps Calories Distance Heart Rate Active Minutes	Water resistant up to 1.5 meters
Yamax (https://www.yamax.co.uk)	SW 200 Digi- walker (AT)	Pedometer	Approx. 3 years	Clip (waist)	0.5 × 0.38 × 0.14 cm	$26.62	Steps	Not Reported

AT: Activity Tracker, SW: Smartwatch
